# Validating a Genomic Convergence and Network Analysis Approach Using Association Analysis of Identified Candidate Genes in Alzheimer’s Disease

**DOI:** 10.3389/fgene.2021.722221

**Published:** 2021-12-09

**Authors:** Puneet Talwar, Suman Kushwaha, Chitra Rawat, Harpreet Kaur, Ankit Srivastava, Rachna Agarwal, Puneet Chandna, Paolo Tucci, Luciano Saso, Ritushree Kukreti

**Affiliations:** ^1^ Genomics and Molecular Medicine Unit, Institute of Genomics and Integrative Biology (IGIB), Council of Scientific and Industrial Research (CSIR), Delhi, India; ^2^ Institute of Human Behaviour and Allied Sciences (IHBAS), Delhi, India; ^3^ Academy of Scientific and Innovative Research (AcSIR), Council of Scientific and Industrial Research (CSIR), Delhi, India; ^4^ Genomic Medicine Institute, Lerner Research Institute, Cleveland Clinic Foundation, Cleveland, OH, United States; ^5^ Department of Pharmacology, Faculty of Pharmacy, Jamia Hamdard, Delhi, India; ^6^ AceProbe Technologies (India) Pvt. Ltd, Delhi, India; ^7^ Department of Clinical and Experimental Medicine, University of Foggia, Foggia, Italy; ^8^ Department of Physiology and Pharmacology “Vittorio Erspamer” Sapienza University, Rome, Italy

**Keywords:** Alzheimer’s disease, genetics, single-nucleotide polymorphism (SNP), APOE, EGFR, ACTB

## Abstract

Previously, we demonstrated an integrated genomic convergence and network analysis approach to identify the candidate genes associated with the complex neurodegenerative disorder, Alzheimer’s disease (AD). Here, we performed a pilot study to validate the *in silico* approach by studying the association of genetic variants from three identified critical genes, *APOE*, *EGFR*, and *ACTB*, with AD. A total of 103 patients with AD and 146 healthy controls were recruited. A total of 46 single-nucleotide polymorphisms (SNPs) spanning the three genes were genotyped, of which only 19 SNPs were included in the final analyses after excluding non-polymorphic and Hardy–Weinberg equilibrium-violating SNPs. Apart from our previously reported *APOE* ε4, four other SNPs in *APOE* (rs405509, rs7259620, −rs769449, and rs7256173), one in *EGFR* (rs6970262), and one in *ACTB* (rs852423) showed a significant association with AD (*p* < 0.05). Our results validate the reliability of genomic convergence and network analysis approach in identifying the AD-associated candidate genes.

## Introduction

Alzheimer’s disease (AD), the most common cause of dementia, is becoming a major health burden worldwide among the elderly due to the aging population, absence of early diagnostic markers, and the lack of disease-modifying treatment (Rajasekhar and Govindaraju, 2018). Globally, 46.8 million were affected by AD in 2015, which is estimated to reach 131.5 million by 2050. As per the Alzheimer’s’ Disease International (ADI) report, Asia Pacific region would witness rise in patients with AD (PwAD) from 23 million in 2015 to 71 million with India having 12 million cases (Prince, 2015).

AD is heritable, but genetically complex. The disease, genetically, is segregated into two forms: familial AD and sporadic, which are clinically indistinguishable. The major genetic signatures identified for familial AD are present in the three genes, namely, the amyloid precursor protein (*APP*) and the presenilins (*PSEN1* and *PSEN2*), while sporadic AD involves the contribution of both genetic and environmental factors (Bekris et al., 2010). However, despite several decades of genetic research, only a small fraction of the genetic variants associated with AD risk have been identified (Wightman et al., 2021). Currently, at least 29 major risk loci for late onset or sporadic AD risk have been uncovered by various large-scale meta-analysis studies (Jansen et al., 2019; Wightman et al., 2021). A major issue with the findings of the genome level studies is the identification of signatures mostly in the non-coding regions with unknown significance. Furthermore, the common variants fail to fully explain the underlying pathophysiology probably due to small effect size (Van Cauwenberghe et al., 2016; Visscher et al., 2021).

As the vast literature is available on genomic studies involving different methodologies such as genome-wide studies including linkage, association, and expression (Talwar et al., 2016), previously, we employed an integrative approach combining multiple data sources along with network modeling of protein–protein interactions to identify the candidate genes associated with AD which revealed *APOE*, *EFGR*, and *ACTB* as the hub genes (Talwar et al., 2014). The aim of the present cross-sectional study was to validate the findings of our genomic convergence and network analysis approach by investigating the association of genetic variants from the three genes with AD in North Indian population.

## Methods

### Study Participants and Phenotyping for Case–Control Study

Individuals of age >50 years and North Indian ancestry attending outpatient neurobehavioral clinic at the Institute of Human Behavior and Allied Sciences (IHBAS) from 2010 to 2012 were enrolled in this cross-sectional study. Family members gave written informed consent, and the study was approved by the Institutional Ethics Committee. The other details related to diagnosis, assessment, investigations, and recruitment of the patients with AD (PwAD) and healthy controls are provided in Supplementary File S1, Methods. The demographic and clinical characteristics of the recruited PwAD as well as healthy controls were reported previously (Talwar et al., 2017).

### Genotyping

Genomic DNA was isolated from the peripheral blood leukocytes using a modification of a salting out procedure (Miller et al., 1988). The functionally important single-nucleotide polymorphisms (SNPs) in *APOE*, *EGFR*, and *ACTB* genes were screened from the literature, and then the functional significance of the SNPs was determined by screening databases including HaploReg, RegulomeDb, rVarBase, and Braineac (in build *in vitro* and *in vivo* evidence). Please refer to Supplementary File S2 for more details. The selected SNPs were genotyped by AceProbe Technologies (India) Pvt. Ltd. by using the Sequenom MassARRAY iPLEX platform (Sequenom Inc., San Diego, United States). The list of primers used is provided in Supplementary File S1, Supplementary Table S1.

### Statistical Analysis

The SNP association analysis was conducted in PLINK version 1.09 (Purcell et al., 2007) and gPLINK (https://zzz.bwh.harvard.edu/plink/gplink.shtml). The imputation, quality control (QC), and association analysis procedure are described in Supplementary File S1, Methods. In brief, after imputation and QC, univariate SNP association analysis was carried out by 2×2 contingency table of χ2 test or Fisher’s exact test. The Benjamini–Hochberg (BH) method was used for multiple testing corrections based on the false discovery rate (FDR) (Benjamini and Hochberg, 1995; Clarke et al., 2011).

Logistic regression analysis was conducted to assess the differences of genotype frequencies between AD and non-demented control groups. The additive model, the dominant model and the recessive model were used in the logistic regression analysis for SNP association with disease phenotype adjusting for age, gender, and education status as covariates. In the additive model, homozygotes for the major allele, and heterozygotes and homozygotes for the minor allele were coded to a quantitative numeric variable for genotypes (0, 1, and 2), implying the additive effects of allele dosage. The dominant and recessive models assume the full dominance [genotype coding (0, 1, and 1)] or recessive [genotype coding (0, 0, and 1)] for the minor allele (Purcell et al., 2007; Laird and Lange, 2011). The linkage disequilibrium (LD) pattern was analyzed using Haploview software (Barrett, 2009). The level of significance was set to 0.05.

## Results

### Basic Characteristics of Study Subjects

A total of 108 PwAD (60.2% men) fulfilling the selection criteria along with 159 healthy controls (51.6% men) of similar ethnicity were enrolled (Talwar et al., 2017). A significant difference was observed in the mean age and education status between the cases and controls (*p* < 0.05) (Talwar et al., 2017), requiring an adjustment of the two potential confounders during the association analysis.

### Frequency Distribution of Genetic Variants

A total of 46 functional SNPs, 10 from *APOE*, 24 from *EGFR*, and 12 from *ACTB* were selected for the case–control association analysis. Out of the total 267 individuals, 12 were excluded either due to the lack of phenotypic data or insufficient DNA and 6 due to the failure of genotyping, resulting in data from 249 total individuals (103 PwAD and 146 controls). Of the 46 SNPs, 12 were found to be non-polymorphic in individuals from the two groups, while 15 failed the Hardy–Weinberg equilibrium (HWE) test in the controls (Supplementary Files S3a,b). The remaining 19 SNPs were included in the final analyses. Among these 19 SNPs, such as *APOE* rs429358 and rs7412, resulted in ε2 (rs429358, T; rs7412, T), ε3 (rs429358, T; rs7412, C), and ε4 (rs429358, C; rs7412, C) haplotypes. We previously reported ε3 to be the most common in both cases and controls, while the presence of the ε4 allele increased the risk of having AD (Talwar et al., 2017).

### Allelic and Genotypic Associations

The allelic and genotypic distribution of 19 SNPs from the three genes in the genotyped 249 samples was evaluated for the association with AD (Supplementary File S4a). Apart from the previously reported *APOE* ε alleles, three other SNPs, namely, rs405509, rs7259620, and rs769449, from *APOE*, and rs6970262 from *EGFR* showed a significant association in genotypic, Cochran–Armitage trend and allelic analysis (*p* < 0.05) (Table 1). None of the genotyped SNPs from the *ACTB* gene showed a significant allelic association. The data for significant associations are provided in Table 1 and for all 19 SNPs in Supplementary Table 4a.

**TABLE 1:** Association analysis of *APOE*, *EGFR*, and *ACTB* SNPs with AD risk among PwAD (*N* = 103) and non-demented controls (*N* = 146).

Gene	SNP	Location	Chr	MAF	Nucleotide change	P_*GENO*_	P_*TREND*_	FDR_*TREND*_	P_*ALLELIC*_	FDR_*ALLELIC*_	P_*DOM*_	FDR_*DOM*_	P_*REC*_	FDR_*REC*_
*APOE*	rs405509	Promoter	19	0.45	g.4798T > G	**0.006**	**0.003**	**0.013**	**0.005**	**0.020**	**0.002**	0.013	0.133	0.399
	rs7259620	Promoter	19	0.41	g.3750G > A	**0.008**	**0.002**	**0.013**	**0.002**	**0.015**	**0.003**	0.013	0.065	0.292
	rs769449	Intron 2	19	0.09	g.5964G > A	3.1E-07*	**1.4E-07**	**2.60E-06**	**3.4E-08**	**6.5E-07**	2.2E-07*	**4.2E-06**	0.028*	0.169
*EGFR*	rs6970262	Intron 21	7	0.076	g.178039A > G	0.051*	**0.018**	0.067	**0.005**	**0.020**	0.069*	0.264	0.036*	0.169
*ACTB*	rs852423	Intron 3	7	0.45	g.6867A > G	**0.041**	0.060	0.190	0.059	0.159	0.496	0.496	**0.012**	0.106

SNP: single-nucleotide polymorphism identifier; Chr; chromosome; MAF: minor allele frequency; GENO: genotypic association; TREND: Cochran–Armitage trend; DOM: dominant model; REC: recessive model; FDR: Benjamini and Hochberg corrected *p* values; P: asymptotic *p* values calculated under chi square test statistic; *p values calculated under fisher exact test; Bold signifies *p* < 0.05.

Logistic regression analysis performed for genotypic association analysis after adjustment for age, gender, and education status showed a significant association of rs405509 (P_additive_ = 0.003, OR = 0.51, 95% CI = 0.33–0.79 and P_dom_ = 0.003, and OR = 0.39, 95% CI = 0.21–0.73), rs7259620 (P_additive_ = 0.007, OR = 0.56, 95% CI = 0.37–0.85 and P_dom_ = 0.005, OR = 0.44, 95% CI = 0.25–0.79), rs769449 (P_additive_ = 2.9E-06, OR = 9.75, 95% CI = 3.76–25.30 and P_dom_ = 1.2E-06, OR = 10.48, 95% CI = 3.98–27.59), and rs7256173 (P_additive_ = 0.021, OR = 5.54, 95% CI = 1.30–23.69 and P_dom_ = 0.021, OR = 5.54, 95% CI = 1.30–23.69) from *APOE.* In *EGFR*, rs6970262 showed significant association only in the additive model (P_additive_ = 0.030, OR = 2.18, 95% CI = 1.08–4.41), and in case of *ACTB*, rs852423 showed significant association only in the recessive model (P_recessive_ = 0.032, OR = 2.18, 95% CI = 1.07–4.46) with AD risk after adjustment for age, gender, and education status. The data for significant associations are provided in Table 2 and for all 19 SNPs in Supplementary Table S4b. Furthermore, rs769449 in *APOE* was found to be in a strong degree of LD with rs429358 (R^2^ = 0.96) and rs7259620 was in LD with rs405509 (R^2^ = 0.88). Despite this, we did not observe the formation of any haplotype block in the study.

**TABLE 2:** Association of *APOE*, *EGFR*, and *ACTB* SNPs with Alzheimer’s disease under logistic regression analysis.

Gene	SNP		Genotype, *N*			A1	P_*unadjusted*_ (*N* = 249)	OR (95%CI)	P_*adjusted*_ additive model (*N* = 237)	OR (95%CI)	P_*adjusted*_ dominant model (*N* = 237)	OR (95%CI)
*APOE*			TT	TG	GG							
	rs405509	N_AD_ N_NDC_	39 29	51 88	13 29	G	**0.003**	0.55 (0.37–0.82)	**0.003**	0.51 (0.33–0.79)	**0.003**	0.39 (0.21–0.73)
			GG	GA	AA							
	rs7259620	N_AD_ N_NDC_	47 40	44 76	12 30	A	**0.003**	0.56 (0.38–0.82)	**0.007**	0.56 (0.37–0.85)	**0.005**	0.44 (0.25–0.79)
			GG	GA	AA							
	rs769449	N_AD_ N_NDC_	71 137	28 9	4 0	A	**2.8E-06**	6.36 (2.93–13.80)	**2.9E-06**	9.75 (3.76–25.30)	**1.2E-06**	10.48 (3.98–27.59)
			CC	CT	TT							
	rs7256173	N_AD_ N_NDC_	96 142	7 4	0 0	T	0.138	2.59 (0.74–9.08)	**0.021**	5.54 (1.30–23.69)	**0.021**	5.54 (1.30–23.69)
*EGFR*			AA	AG	GG							
	rs6970262	N_AD_ N_NDC_	7 2	10 10	86 134	A	**0.023**	1.98 (1.10–3.58)	**0.030**	2.18 (1.08–4.41)	0.060	2.34 (0.97–5.65)

OR: odds ratio; CI: confidence interval; A1: minor/effect allele; N_AD_: Genotypes number in AD; N_NDC_: Genotypes number in non-demented controls; P _*adjusted*_: *p* values adjusted with age, gender and education status in logistic regression analysis. Bold signifies *p* < 0.05. Additive model is based on the additive effects of allele dosage. Dominant model assumes the full dominance for the minor allele. The OR > 1, means A1 increases relative risk relative to A2. All values are calculated using PLINK, 1.09.

## Discussion

In the present study, we conducted a pilot genetic association analysis comprising 249 study subjects to validate the findings of our genomic convergence and network analysis using the *in silico* approach. A total of 46 SNPs in the three identified hub genes, namely, *APOE*, *EGFR*, and *ACTB*, were screened for functional genetic variants to perform the association study. The study observed a significant association of six SNPs with AD: rs405509, rs7259620, rs769449, and rs7256173 from *APOE*, rs6970262 from *EGFR*, and rs852423 from *ACTB*. Despite strong LD between the SNPs of the *APOE* gene, we did not observe a haplotype block in the studied cohort. This could potentially be due to the presence of multiple SNPs in LD, where uneven distance makes it difficult to determine the exact boundaries of the haplotype block (Takeuchi et al., 2005).

*APOE* is the most widely studied gene with respect to AD genetics due to its high abundance in different brain cells (Lefterov et al., 2019). Published reports indicate that *APOE* polymorphisms are associated with AD pathophysiology involving the plaque deposition of intraneuronal amyloid-β (Aβ) aggregates, and hyperphosphorylated tau-mediated neurodegeneration (Yamazaki et al., 2019). In addition, peripheral ApoE also plays a potential role in lipid homeostasis in the brain (Martinez-Morillo et al., 2014). We previously reported the *APOE* ε4 allele to be associated with increased susceptibility for AD (Talwar et al., 2017). In the current study, apart from the epsilon alleles, we reported other *APOE* promoters and intronic SNPs including rs405509, rs7259620, rs769449, and rs7256173 to be associated with AD. The rs405509 TT genotype was observed to be over-represented in PwAD. In a previous case–control study, the rs405509 “T” homozygote was associated with the increased risk for developing AD (Lambert et al., 2002). The same group also reported that the patients with the rs405509 TT genotype had higher levels of total Aβ in the Brodmann area of the cerebral cortex than those with GT/GG genotypes (Lambert et al., 2001), indicating the involvement of rs405509 in the processing of β amyloid. Individuals homozygous for the “T” allele were also reported to have higher number of senile plaque in hippocampus CA1 and the subiculum (Berr et al., 2001), and the elderlies displayed cognitive impairment and low gray matter volume (Ma et al., 2016). Such effects could be due to lower brain ApoE levels in individuals with the TT genotype at rs405509. Substitution of rs405509 “T” to the minor allele “G” significantly increases the *APOE* promoter activity (Artiga et al., 1998), suggesting a protective effect of the rs405509 “G” allele in AD, as observed in our study. These findings provide evidence that rs405509 may be involved in the *APOE* gene regulation and, therefore, may serve as a potential biomarker in AD.

The association of another *APOE* promoter SNP, rs7259620, in the present study was supported by similar findings from a case–control study involving Japanese population where the minor allele “A” was observed to have a protective role in LOAD (Takei et al., 2009); however, its role in *APOE* regulation is still unexplored. We also found the intronic *APOE* SNP, rs769449, to be highly associated with AD, where the presence of “A” allele showed an increased risk. The SNP was previously reported to be associated with a cognitive decline among European-Americans and African-Americans (Zhang and Pierce, 2014). Cruchaga et al. (2013) observed rs769449 to be associated with the CSF levels of tau and ptau proteins, two key biochemical markers of axonal degeneration, neuronal loss, and a cognitive decline in PwAD, suggesting a role of this intronic SNP in AD pathophysiology. Nevertheless, the effect of “G” to “A” substitution in this SNP has not been investigated yet. Last, the *APOE* genetic variant, rs7256173, has not been previously associated with AD.

Another gene, *EGFR*, encodes the epidermal growth factor receptor, a transmembrane protein that binds to the epidermal growth factor. *EGFR* knock-out mice were reported to develop neurodegeneration leading to early death due to defects in cortical neurogenesis, indicating that *EGFR* signaling plays a role in neurogenesis (Wong and Guillaud, 2004). Our study identified “A” allele/AA genotype of the *EGFR* intronic variant rs6970262 to be significantly associated with increased AD risk.

Although rs6970262 has not been previously associated with AD, previous investigations from our group reported *EGFR* as a potential candidate for assessing AD risk (Talwar et al., 2014; Talwar et al., 2017). The EGFR was indicated as a factor that mediates beta amyloid (Aβ) in animal models (Wang et al., 2012). Moreover, poor olfactory discrimination associated with AD may be due to the reduced EGF-dependent olfactory neurogenesis (Enwere et al., 2004). The olfactory system is a relevant effector in different animal models of neurodegenerative diseases (Loseva et al., 2009). Moreover, the olfactory bulbectomy is a known animal model of depression (Song and Leonard, 2005), and the neuroimaging studies found reduced olfactory bulb volume in depressive patients (Negoias et al., 2010). Depression is a risk factor for AD (Modrego and Ferrández, 2004; Ownby et al., 2006; Sun et al., 2008) and frequently observed in preclinical AD (Geerlings et al., 2000; Visser et al., 2000). It has been reported that soluble Aβ induces a depressive-like phenotype in rats (Colaianna et al., 2010). Hence, considering that a pilot study has found a possible relationship between depression and *EGFR* mutation status in patients with non–small-cell lung cancer (Jacobs et al., 2017), the *EGFR* intronic variant rs6970262 could be useful to characterize depressed patients with high risk to develop AD.

The presence of immunoreactive EGFR was also observed in neuritic plaques from PwAD (Birecree et al., 1988). Similar findings were reported in the brain vasculature of demented elderly patients where the increased EGFR expression was related to proliferative or regenerative activities in the vascular architecture of PwAD, suggesting that it might be used as a potential biomarker for early diagnosis of dementia using skin biopsy (Styren et al., 1990; Styren et al., 1993).

Our study also involved important SNPs from the gene *ACTB*, which encodes the non-muscle cytoskeletal actin, β-actin. Mutations in this gene cause Baraitser–Winter syndrome 1 and juvenile-onset dystonia (https://www.genecards.org/cgi-bin/carddisp.pl?gene=ACTB, last update: 24 May 2021). Due to the highly conserved sequence and ubiquitous expression of *ACTB*, the gene is used as a reference for the normalization of target gene expression in mRNA and protein studies. However, Leduc et al. (2011) found the gene to be an unsuitable reference gene due to lower stability of mRNA in the frontal cortex of PwAD, suggesting dysregulation of the gene in AD. In addition, an integrated *in silico* analysis involving four different gene expression datasets also found *ACTB* to be a central gene in AD pathophysiology (Hu et al., 2015). Our study observed a significant genotypic association of the *ACTB* variant rs852423 with increased susceptibility to AD. The association of this SNP with AD also appears in the signaling network presented in the database NeuroMMSig (Domingo-Fernández et al., 2017).

The current knowledge is limited, but a connection from this gene and glucose metabolism in AD pathophysiology is worth to be explored in the future studies. In fact, *in vitro* experiment that generated insulin-secreting beta cells from human pluripotent stem cells has shown the fundamental role of actin in pancreatic progenitor gene expression and endocrine function (Hogrebe et al., 2020; Siehler et al., 2020). The pancreas has a central role in the glucose metabolism, and the glycolytic dysfunction has been associated with the dysfunction of Alzheimer’s brains, such as synaptic impairment, brain atrophy, mitochondrial impairment, and Aβ deposition (Zhang et al., 2021). Moreover, diabetes has been classified as a risk factor for AD (Profenno et al., 2010), especially when associated with the *APOE ε4* genotype. The subjects carrying an *APOE ε4* allele without dementia have shown a reduction in the cerebral metabolic rate of glucose (Mosconi et al., 2008).

The present study has some inherent limitations such as the low sample size. However, our study was a pilot investigation to validate the findings of our *in silico* approach (Talwar et al., 2014), and therefore, a small sample size may yield a greater scientific value relative to the expense of the genotyping required to perform the study (Bacchetti et al., 2011). In addition, our study performed the association analysis with very few genes, in which the role of potentially actionable novel targets may get overlooked. Therefore, to establish the robustness of our *in silico* findings, future studies with larger sample sizes using an expanded gene list need to be performed.

In summary, this study provides the first evidence of the association of the *EGFR* genetic variant rs6970262. Our previous *in silico* analysis (Talwar et al., 2014) and its validation in clinical samples in the current study provide evidence of the association of regulatory variants present in the promoter and intronic regions of *APOE*, *EGFR*, and *ACTB*, highlighting the importance of integration of experimental and computational approaches to reveal the clinical significance of genetic variants in a disease phenotype. However, as multiple SNPs are implicated in complex disorders such as AD, a combination of genetics with biochemical serum markers would be crucial to uncover the pathophysiological cascade (Talwar et al., 2014; Talwar et al., 2017). Our results may help to reveal the functional role of these variants in the pathogenesis of AD. Further validation studies are required to confirm our findings and elucidate the mechanistic role of these polymorphisms in AD pathophysiology.

## Data Availability

The raw data supporting the conclusion of this article will be made available by the authors, without undue reservation.
