# Supplementary material for: Validating a Genomic Convergence and Network Analysis Approach Using Association Analysis of Identified Candidate Genes in Alzheimer’s Disease
Source: Front Genet. 2021 Dec 9;12:722221. doi: 10.3389/fgene.2021.722221 (PMC8696000; doi:10.3389/fgene.2021.722221)
Supplement: Supplementary file 1 [file DataSheet1.docx]

# Validating a genomic convergence and network analysis approach using association analysis of identified candidate genes in Alzheimer's disease

Puneet Talwar^1,2^,Suman Kushwaha^2^,Chitra Rawat^1,3,#^,Harpreet Kaur^4,#^,Ankit Srivastava^1,5^, Rachna Agarwal^2^, Puneet Chandna^6^, Paolo Tucci^7^, Luciano Saso^8^, Ritushree Kukreti^1,3,*^

^1^Genomics and Molecular Medicine Unit, Institute of Genomics and Integrative Biology (IGIB), Council of Scientific and Industrial Research (CSIR), Delhi, India

^2^Institute of Human Behaviour & Allied Sciences (IHBAS), Dilshad Garden, Delhi 110095, India

^3^Academy of Scientific and Innovative Research (AcSIR), Council of Scientific and Industrial Research (CSIR), Mall Road, Delhi-110007, India

^4^Genomic Medicine Institute, Lerner Research Institute, Cleveland Clinic Foundation, 9500 Euclid Avenue, Cleveland, Ohio 44195, USA

^5^Department of Pharmacology, Faculty of Pharmacy, Jamia Hamdard, Delhi-110062, India

^6^AceProbe Technologies (India) Pvt.Ltd

^7^Department of Clinical and Experimental Medicine, University of Foggia, Italy

^8^Department of Physiology and Pharmacology "VittorioErspamer" Sapienza University, P.le Aldo Moro 5, 00185, Rome, Italy

**Correspondence:** Ritushree Kukreti, Genomics and Molecular Medicine Unit, IGIB (CSIR), Mall Road, Delhi 110 007, India, Phone: 91-11-27662201; Fax: 91-11-27667471; Email:[ritus@igib.res.in](mailto:ritus@igib.res.in);ORCID ID:<https://orcid.org/0000-0002-6968-1129>

**^#^These authors made equal contributions to the work.**

# Methods

**Study participants and phenotyping for case-control study**

All the subjects underwent assessment for cognitive decline through Mini-Mental State examination (MMSE) which was administered by a trained resident doctor. The MMSE scale is one of the most commonly and widely used assessment tool to screen for cognitive impairment, categorizing patients, according to National Institute for Health and Care Excellence guidance, as severely impaired (<10 points), moderately impaired (10–20 points), or mildly impaired (21–26 points). The MMSE measures temporal and physical orientation, declarative memory, language, working memory, and motor/constructional function. Based on the MMSE data, subjects with MMSE score less than 26 were subsequently examined by a experienced neurologist. All patients underwent routine blood tests and brain imaging. The National Institute of Neurological and Communicative Disorders Association-Alzheimer’s Disease and Related Disorders Association (NINCDS-ADRDA) criteria was used for establishing the diagnosis of probable AD. The diagnosis was confirmed by CT head or MRI brain studies, which showed a generalized atrophy with selective temporo-parietal atrophy in all the patients.

Healthy individuals accompanying the AD patients or from community having age ≥ 50 and North Indian decent without the presence of neurological or psychiatric disorder, no family history of neurological or psychiatric disorder and no other debilitating medical conditions were enrolled. All controls were recruited after evaluating clinical history and examination which includes the mini–mental status examination (MMSE) and other higher mental functions. Individuals with MMSE score > 27 were selected for the study. Habitual smoker or alcoholic or individuals with history of substance abuse were excluded from the study.

**Statistical analysis**

The SNP association analysis was conducted in PLINK version 1.09 and gPLINK. fastPHASE was used for imputation of missing genotypes. The input file for fastPHASE was created through fcGene software using PLINK .ped and .map files. PLINK QC procedures for genotype data cleaning were performed to identify poor quality or otherwise questionable SNPs. In the QC, the quality for samples was checked excluding individuals with > 10% missing genotype data (genotyping rate <90%) also termed as missing call rate (MCR, set at 0.10); SNPs that have <1% minor allele frequencies (MAF); Hardy-Weinberg equilibrium (HWE) set at P<0.001; and sex differences according to allelic frequency or heterozygosity rate.

Single SNP association to disease was evaluated by chi square test or Fisher’s Exact test and results were confirmed in Haploview. Linkage disequilibrium (LD) analysis was conducted with the Haploview software. Benjamini-Hochberg (BH) method was used for multiple testing correction based on false discovery rate (FDR). Using PLINK, a logistic regression analysis assuming additive, dominant model and recessive model was used for SNP association analysis adjusting for age, gender and education status as covariates. The level of significance was set to 0.05.The output of single SNP association analysis in PLINK yielded results of five test namely GENO (genotypic association), TREND (Cochran-Armitage trend), Allelic (allelic association), DOM (dominant model) and REC (recessive model). The result output is provided in Supplementary file 4a. For logistic regression analysis, --logistic command in PLINK was used with and without covariates (age, gender and education status) for additive model. For dominant and recessive model analysis in logic regression --logistic command followed by either –dominant or –recessive was used. The result output is provided in Supplementary file 4b.

**Table S1:** PCR and extension primer sequences for genotyping SNP (performed on a matrix-assisted laser desorption/ionization (MALDI)-TOF mass-spectrometer (MassArray system) employing the Spectro designer software (Sequenom, CA, USA)

| **S.No.** | **Gene** | **db SNP Id** | **Genomic Location** | **Forward Primer (FP) (5’→3’)** | **Reverse Primer (RP) (5’→3’)** | **Extension Primer (5’→3’)** |
| --- | --- | --- | --- | --- | --- | --- |
| 1 | *APOE* | rs7256173 | chr19:44904398 | ACGTTGGATGCTTGCCAGCCAAAGCAAACA | ACGTTGGATGTGAACTCCTGGTTCGAGAAG | ccGGAACCCCCAGTGCC |
| 2 | *APOE* | rs7259620 | chr19:44904531 | ACGTTGGATGAATGAGTCCCAGTCTCTCCC | ACGTTGGATGTTTCAGAGGAGAAACCCGTG | ggattTGTGGTTTTGCCATTC |
| 3 | *APOE* | rs769446 | chr19:44905371 | ACGTTGGATGCTTAAGTGATTCGCCCACTG | ACGTTGGATGGCTGGACAGAAGTGGGATG | ATTACAGGCGTGAGC |
| 4 | *APOE* | rs405509 | chr19:44905579 | ACGTTGGATGACATTCCCCTTCCACGCTTG | ACGTTGGATGTTTTGACCACCCCCCACAGT | GGAGGAGGGTGTCTG |
| 5 | *APOE* | rs440446 | chr19:44905910 | ACGTTGGATGCCTCTAGAAAGAGCTGGGAC | ACGTTGGATGTAGCTCTCCTGAGACTACCT | ggaaTACCTGGAGGCCAGGG |
| 6 | *APOE* | rs143063029 | chr19:44906731 | ACGTTGGATGTCCTCCCCCTCTCATCCTCA | ACGTTGGATGACAGAAGCCTCAGAAGAGGG | ggtagGCCTGAATGGGGCCA |
| 7 | *APOE* | rs769449 | chr19:44906745 | ACGTTGGATGTCTCATCCTCACCTCAACCT | ACGTTGGATGTCAAATCGCTGTTCAGAGCC | CCTGGCCCCATTCAG |
| 8 | *APOE* | rs429358 | chr19:44908684 | ACGTTGGATGCTGTCCAAGGAGCTGCAGG | ACGTTGGATGTGCACCTCGCCGCGGTACT | tGACATGGAGGACGTG |
| 9 | *APOE* | rs7412 | chr19:44908822 | ACGTTGGATGACCTGCGCAAGCTGCGTAA | ACGTTGGATGGCCCCGGCCTGGTACACTG | ccgtCCTGGTACACTGCCAGGC |
| 10 | *APOE* | rs769450 | chr19:44907187 | ACGTTGGATGATCTGCCCGTTTCGATCTCC | ACGTTGGATGGCCTGCAATGCATTAGAAAC | CGCACCTGGCTGGGA |
|  |  |  |  |  |  |  |
| 11 | *ACTB* | rs281875334 | chr7:5528496 | ACGTTGGATGTCTCCTTAATGTCACGCACG | ACGTTGGATGTGACTGACTACCTCATGAAG | TGGTGAAGCTGTAGCCG |
| 12 | *ACTB* | rs104894003 | chr7:5528536 | ACGTTGGATGTGGTGGTGAAGCTGTAGCC | ACGTTGGATGCCATGCCATCCTGCGTCTG | tcCTGGACCTGGCTGGC |
| 13 | *ACTB* | rs397515470 | chr7:5529175 | ACGTTGGATGAAGAGGTAGCGGGCCACTCA | ACGTTGGATGAGCACCCCGTGCTGCTGAC | CTCACCTGGGTCATCTTCT |
| 14 | *ACTB* | rs281875332 | chr7:5529331 | ACGTTGGATGATGTCGTCCCAGTTGGTGA | ACGTTGGATGAAGGATTCCTATGTGGGCGA | gGAGCAAGAGAGGCATC |
| 15 | *ACTB* | rs281875331 | chr7:5529624 | ACGTTGGATGCGAAGCCGGCCTTGCACAT | ACGTTGGATGCTATTCTCGCAGCTCACCAT | ACATGCCGGAGCCGT |
| 16 | *ACTB* | rs2966449 | chr7:5531994 | ACGTTGGATGTCTTTGCAGCGCGTAGTAGG | ACGTTGGATGCCATGTCCTTATATGGACTC | GCGTAGTAGGTGTTCA |
| 17 | *ACTB* | rs11546899 | chr7:5528355 | ACGTTGGATGTTCGAGCAAGAGATGGCCAC | ACGTTGGATGACCGCTCATTGCCAATGGTG | GAAGAGCTACGAGCTGC |
| 18 | *ACTB* | rs7612 | chr7:5527481 | ACGTTGGATGTGGCCGAGGACTTTGATTGC | ACGTTGGATGCTTTTAGGATGGCAAGGGAC | TCATTCCAAATATGAGATGC |
| 19 | *ACTB* | rs852425 | chr7:5527040 | ACGTTGGATGAAGGCTGCTCAATGTCAAGG | ACGTTGGATGTGAACCCTGCAAAAGGGTGG | ggaaACACACCTGCACTCTGGGT |
| 20 | *ACTB* | rs852423 | chr7:5528735 | ACGTTGGATGAGTGGCTTCCCCAGTGTGAC | ACGTTGGATGCTGGATAGCAACGTACATGG | CCCCAGTGTGACATGGTG |
| 21 | *ACTB* | rs852392 | chr7:5532501 | ACGTTGGATGTTCGTGGAACGTTGAACTGG | ACGTTGGATGACCTGACAACCTCTCATCCC | GGGGATGGGACCGGATCT |
| 22 | *ACTB* | rs852391 | chr7:5532595 | ACGTTGGATGTGCAGAGCAGACCTGATCC | ACGTTGGATGGTCTCTTGGCCAGCTGAATG | ctGCAGGACCTGTGTTGCC |
|  |  |  |  |  |  |  |
| 23 | *EGFR* | rs28929495 | chr7:55174014 | ACGTTGGATGTCTCTTGAGGATCTTGAAGG | ACGTTGGATGTTACCTTATACACCGTGCCG | CAAAAAGATCAAAGTGCTG |
| 24 | *EGFR* | rs121913428 | chr7:55174015 | ACGTTGGATGTCTCTTGAGGATCTTGAAGG | ACGTTGGATGTTACCTTATACACCGTGCCG | ccgttCCGAACGCACCGGAG |
| 25 | *EGFR* | rs121913446 | chr7:55174735 | ACGTTGGATGTCTCTCTGTCATAGGGACTC | ACGTTGGATGAATTCCTTGATAGCGACGGG | GGAATTTTAACTTTCTCACCTTCT |
| 26 | *EGFR* | rs121913432 | chr7:55181327 | ACGTTGGATGTCCAGGAAGCCTACGTGATG | ACGTTGGATGATGAGCTGCGTGATGAGCTG | ccCCAGCGTGGACAACCCCC |
| 27 | *EGFR* | rs121434569 | chr7:55181378 | ACGTTGGATGATCTGCCTCACCTCCACCGT | ACGTTGGATGTGTTCCCGGACATAGTCCAG | CCGTGCAGCTCATCA |

**Table S1: (continued)**

| **S.No.** | **Gene** | **db SNP Id** | **Genomic Location** | **Forward Primer (FP) (5’→3’)** | **Reverse Primer (RP) (5’→3’)** | **Extension Primer (5’→3’)** |
| --- | --- | --- | --- | --- | --- | --- |
| 28 | *EGFR* | rs397517127 | chr7:55191749 | ACGTTGGATGATGAACTACTTGGAGGACCG | ACGTTGGATGTGTGATCTTGACATGCTGCG | TTGGAGGACCGTCGCTTG |
| 29 | *EGFR* | rs2072454 | chr7:55146655 | ACGTTGGATGTGCGGTTCAGCAACAACCCT | ACGTTGGATGAGTCACTGCTGACTATGTCC | CCCTGCCCTGTGCAA |
| 30 | *EGFR* | rs1050171 | chr7:55181370 | ACGTTGGATGTGTTCCCGGACATAGTCCAG | ACGTTGGATGTGCTGGGCATCTGCCTCAC | GAGCTGCGTGATGAG |
| 31 | *EGFR* | rs9642393 | chr7:55177954 | ACGTTGGATGTTTAGCTTCTGCGTACACCG | ACGTTGGATGATCTGATAGACCCACTGGGC | tcGAACAGCGTTCCCAT |
| 32 | *EGFR* | rs1140475 | chr7:55198724 | ACGTTGGATGATGGCTTGGATCCAAAGGTC | ACGTTGGATGTCATTCATGATCCCACTGCC | GGTCATCAACTCCCAAAC |
| 33 | *EGFR* | rs2227983 | chr7:55161562 | ACGTTGGATGTCTGCCATGCCTTGTGCTC | ACGTTGGATGCTGACATTCCGGCAAGAGAC | gcaGGGGCCCGGAGCCCA |
| 34 | *EGFR* | rs4947986 | chr7:55153962 | ACGTTGGATGTTTCTGACGGGAGTCAACAC | ACGTTGGATGTATGGAGGAGAGAGGACACG | cccgTCCTCCGTGTGTGGC |
| 35 | *EGFR* | rs884419 | chr7:55208587 | ACGTTGGATGTCATCTCTAAGGAGCTCCTC | ACGTTGGATGGAGTTTAGAAGACTCATCCC | aCTCTAATTACACCATGCCC |
| 36 | *EGFR* | rs763317 | chr7:55027504 | ACGTTGGATGCCATGAGGAAACCTGGAATG | ACGTTGGATGTTCACATCTGGTTGCTACTT | GGAAACCTGGAATGTCAAAG |
| 37 | *EGFR* | rs6593205 | chr7:55100999 | ACGTTGGATGGAGGAGAAGAATCAGCTGTC | ACGTTGGATGCCTTTACCTGGTGCTTTTCC | tttgGCACGAGGAGAAACAGT |
| 38 | *EGFR* | rs12535226 | chr7:55088726 | ACGTTGGATGTTGCGAAGGCCTTTCATTCC | ACGTTGGATGCCTTAACCATGTGTCAACCC | CCTTTCATTCCAGAAAGAAAAC |
| 39 | *EGFR* | rs11977660 | chr7:55094643 | ACGTTGGATGCAAAGTCTTGGAGTATCCAG | ACGTTGGATGTTACGACAAGACAGTCCAGC | TGGAGTATCCAGCATATGTGAA |
| 40 | *EGFR* | rs2017000 | chr7:55174916 | ACGTTGGATGGGAGTTATACCCACTAGAGC | ACGTTGGATGAGCTGCTCTGCTCTAGACC | ggGAACATTTAGGATGTGGAGA |
| 41 | *EGFR* | rs845561 | chr7:55185015 | ACGTTGGATGCTTACCTAGATAGTTTGTGCC | ACGTTGGATGTCCTGGCATCCTCAAAATGG | ggagcTAGTTTGTGCCTTTGTGA |
| 42 | *EGFR* | rs712829 | chr7:55019062 | ACGTTGGATGGAGCTAGACGTCCGGGCAG | ACGTTGGATGCGTCGGGCGCTCACACCGT | GCCGCAGCAGCCTCC |
| 43 | *EGFR* | rs884225 | chr7:55206391 | ACGTTGGATGGCTGATTTCATGACAGCAAG | ACGTTGGATGATGAGCGTTAGACTGACTTG | ACAGCAAGACAGGGGC |
| 44 | *EGFR* | rs6970262 | chr7:55192070 | ACGTTGGATGAGGGTCTCCTGGTAGTGTGA | ACGTTGGATGACATCCTCATTCACTGTCCC | ccAGCTGCTTTGGGAAC |
| 45 | *EGFR* | rs4947963 | chr7:55020722 | ACGTTGGATGGACATCAACAGCAAGTACGG | ACGTTGGATGGGGAAACATGCCTTGGAAG | gaGCAGCTCAAGGTTGG |
| 46 | *EGFR* | rs11979158 | chr7:55091656 | ACGTTGGATGCCACTAGTCAGGACCAGATT | ACGTTGGATGCATGTCGAAGCTATTGGTGG | tTCAGGACCAGATTTTAGAA |
